# Mechanisms of activation of mouse and human enteroendocrine cells by nutrients

**DOI:** 10.1136/gutjnl-2014-306834

**Published:** 2014-07-11

**Authors:** Erin L Symonds, Madusha Peiris, Amanda J Page, Bridgette Chia, Harween Dogra, Abigail Masding, Vasileios Galanakis, Michael Atiba, David Bulmer, Richard L Young, L Ashley Blackshaw

**Affiliations:** 1 Nerve-Gut Research Laboratory, Hanson Institute, Royal Adelaide Hospital, Adelaide, South Australia, Australia; 2 Wingate Institute of Neurogastroenterology, Blizard Institute, Barts and The London School of Medicine & Dentistry, Queen Mary, University of London, London, UK; 3 Discipline of Medicine, University of Adelaide, Adelaide, South Australia, Australia

**Keywords:** Obesity, Gut Hormones

## Abstract

**Objective:**

Inhibition of food intake and glucose homeostasis are both promoted when nutrients stimulate enteroendocrine cells (EEC) to release gut hormones. Several specific nutrient receptors may be located on EEC that respond to dietary sugars, amino acids and fatty acids. Bypass surgery for obesity and type II diabetes works by shunting nutrients to the distal gut, where it increases activation of nutrient receptors and mediator release, but cellular mechanisms of activation are largely unknown. We determined which nutrient receptors are expressed in which gut regions and in which cells in mouse and human, how they are associated with different types of EEC, how they are activated leading to hormone and 5-HT release.

**Design and results:**

mRNA expression of 17 nutrient receptors and EEC mediators was assessed by quantitative PCR and found throughout mouse and human gut epithelium. Many species similarities emerged, in particular the dense expression of several receptors in the distal gut. Immunolabelling showed specific colocalisation of receptors with EEC mediators PYY and GLP-1 (L-cells) or 5-HT (enterochromaffin cells). We exposed isolated proximal colonic mucosa to specific nutrients, which recruited signalling pathways within specific EEC extracellular receptor-regulated kinase (p-ERK) and calmodulin kinase II (pCAMKII), as shown by subsequent immunolabelling, and activated release of these mediators. Aromatic amino acids activated both pathways in mouse, but in humans they induced only pCAMKII, which was colocalised mainly with 5-HT expression. Activation was pertussis toxin-sensitive. Fatty acid (C12) potently activated p-ERK in human in all EEC types and evoked potent release of all three mediators.

**Conclusions:**

Specific nutrient receptors associate with distinct activation pathways within EEC. These may provide discrete, complementary pharmacological targets for intervention in obesity and type II diabetes.

**Key messages**

- **What is already known on this subject?**
- Luminal nutrients stimulate enteroendocrine cells (EEC) to release mediators that potently inhibit appetite and promote glucose homeostasis.
- Several specific G-protein-coupled nutrient receptors (GPCR) exist for dietary sugars, amino acids and fatty acids, which may be expressed on EEC.
- Activation of nutrient receptors and mediator release from the distal gut is central to the action of bypass surgery for obesity and type II diabetes, but cellular mechanisms of activation are largely unknown.
- **What are the new findings?**
- Nutrient receptors for fatty acids and amino acids are expressed throughout mouse and human gut epithelium, notably in the large intestine.
- There is specific colocalisation of receptors with specific EEC mediators, for example, calcium-sensing receptors and 5-HT.
- Luminal nutrient exposure activates mediator release and recruits specific GPCR signalling pathways within EEC cells: in humans, tryptophan and phenylalanine activate only pCAMKII in enterochromaffin cells, whereas lauric acid activates p-ERK.
- **How might it impact on clinical practice in the foreseeable future?**
- It is already known that oral nutrient preloads reduce subsequent food intake and that bypass surgery reduces food intake by shunting nutrient to the distal gut. By refining nutrient preloads and formulating them to target the distal gut, we expect to develop a successful weight loss and antidiabetic strategy prior to, and possibly in place of, bypass surgery.

## Introduction

Nutrient sensing in the gut epithelium is fundamental to glucose homeostasis, energy intake, motor and secretory function. Mechanisms of carbohydrate sensing are becoming better understood, but those of protein, amino acid (AA) and fatty acid sensing are less well described, especially in humans. Improved knowledge of the sites and mechanisms of nutrient sensing will ultimately allow us to optimise the control of glycaemia and gastroparesis in type II diabetes, hyperphagia associated with obesity and hypophagia associated with aging.

Nutrients are sensed by enteroendocrine cells (EEC) as breakdown products of carbohydrate, fat and protein digestion. This may occur via activity of the sodium-coupled transporters involved in AA and glucose absorption, which excite the cells and lead to release of mediators such as glucagon-like peptide 1 (GLP-1) in the case of L-cells.^1^ ^2^ Circulating GLP-1 then boosts pancreatic insulin release. L-cells also release peptide YY (PYY), which is important in suppressing appetite. There are several other classes of EECs, one of the most important being the enterochromaffin cell (EC), which releases mainly 5-hydroxytryptamine (5-HT). The role of EC in nutrient sensing is evident in the blockade of sensory^3^ nerve responses to intraluminal glucose by a 5-HT3 receptor antagonist,^4^ but their role in glycaemic and appetite control is unclear. In addition to the transporter-mediated release of gut mediators, there are specific G-protein-coupled receptors (GPCR) for sugars (a dimer of T1R2 and T1R3), and free fatty acids, including FFAR1, 2 and 3 (also known as GPR40, 43 and 41, respectively), GPR84 and GPR120. In some cases, receptors have been found in the gut and colocalised in EECs^5^ with hormones such as PYY and GLP-1.^1^ ^6^ ^7^ Protein breakdown products may also act via a specific receptor, GPR93,^8^ and specific AAs act via calcium-sensing receptors (CaSR), GPRC6A, T1R1 and the metabotropic glutamate receptor mGluR4.^9^ However, very little is known about the expression and colocalisation of these receptors in human gut, in which regions they are found and how this compares with data from other species. Since nutrient absorption is primarily achieved in the small intestine, it is assumed that detection of nutrients and feedback control of energy intake is also mediated there. However, the potent effects of gastric bypass surgery on energy intake and glycaemic control indicate that diverting nutrient to the distal small intestine and large intestine can initiate major effects on energy intake. Moreover, products of primary protein and fat digestion can be found in the normal colon,^3^ and nutrients administered in the distal gut have potent effects on upper gut function.^10^

Bypass surgery is the most effective current intervention in obesity and type II diabetes. By shunting nutrient to distal regions of the intestine, it causes copious postprandial release of satiety and incretin hormones PYY and GLP-1 via chemosensory mechanisms.^11^ There are large and rapid benefits in terms of weight loss and blood glucose homeostasis that coincide with PYY and GLP-1 levels.^12–14^ Improved glycaemia precedes weight loss, but the two are inextricably linked. Despite these advantages, surgical treatments have several drawbacks, including their application in only severe cases due to irreversibility, and cost. Not surprisingly, there is growing interest among gastroenterologists and endocrinologists in developing non-surgical therapies that lead to a reduction in energy intake, weight loss and resolution of type II diabetes. Targeting the initiation of the satiety signal arising from the GI tract is an attractive option. It is also becoming apparent that protein digestion products have potent effects on hormone release and glycaemic control, which may be of therapeutic benefit,^15^ although the mechanism of these actions is currently unclear. Accordingly, we are pursuing the opportunity to intervene directly with fatty acid, AA and protein-sensing pathways of the distal gut to modify endocrine responses.

We hypothesised that nutrient receptor expression is preserved in the distal gut and is able to elicit responses to specific luminal nutrients. We show how patterns of expression of nutrient receptors along the gut are comparable in human and mouse, how they may couple with release of gut hormones from specialised cells in humans and which signalling cascades are responsible. Gleaning this information we hope will reveal a diverse range of targets for future antiobesity treatments that will directly address the aetiology of the condition.

## Methods

Animal studies were approved by the Animal Ethics Committees of the University of Adelaide and Central Northern Adelaide Health Service. All animal procedures in the UK were conducted in compliance with the Home Office guidelines. Human studies were approved by the Human Research Ethics Committees of the Royal Adelaide Hospital or Barts and The London NHS Trust, and were performed with written informed consent.

### Mice

Experiments were performed using adult female C57BL/6 mice maintained on standard laboratory chow and water in a controlled environment (12 h light/dark cycle, 22±0.5°C, 40–60% humidity) within a pathogen-free facility. Mice were killed by CO_2_ asphyxiation. For expression analysis, the following were collected: antrum, small intestine (proximal 2 cm was designated as duodenum, with the remainder divided equally into proximal, mid and distal jejunum and ileum), caecum and large intestine (proximal and distal). Mucosa from each segment was collected by scraping, frozen in liquid N_2_ and stored (−80°C) for later gene expression measurements. For immunohistochemistry, intact tissues were fixed in 4% paraformaldehyde (2 h), cryoprotected and mounted for sectioning. For Ussing chamber experiments, the proximal colon was rapidly removed, opened and pinned flat for 30 min with the mucosa facing up before being placed into the divided Ussing chamber.

### Human tissue collection

Biopsies from histologically normal intestinal mucosa were collected for gene expression studies from fasted subjects undergoing surveillance enteroscopy or colonoscopy. Biopsies were collected from the antrum, duodenum, proximal and mid-jejunum (mean depth 167 cm) and, separately, from the right, transverse and left colon, and rectum and were histologically normal. All biopsies were stored in RNAlater at −20°C (Qiagen) prior to gene expression measurements.

For immunohistochemistry, full-thickness samples of human proximal colon were obtained from patients undergoing surgery for GI cancer. Following surgical resection, non-pathological tissue was fixed in Zamboni's fixative (4°C overnight). Tissues were cryoprotected in 30% sucrose/phosphate buffered saline then mounted in optimum cutting temperature medium.

For Ussing chamber studies, ascending colonic tissue was obtained from patients undergoing right hemicolectomy. Non-pathological full-thickness samples were isolated and placed in cold carbogenated Krebs solution before excision of muscular and serosal layers from the mucosa in a silicone-filled Petri dish. The remaining mucosa was pinned flat facing up before being placed into the Ussing chamber.

Where hormone/peptide release was measured, four ascending colon biopsies/patient were collected from 15 patients. Tissue was immediately placed in cold carbogenated Krebs solution before being transferred to a 96-well plate containing nutrient and control solutions.

### Gene expression studies

Quantitative real-time reverse transcriptase PCR (RT-PCR) was used to assess relative expression of nutrient GPCRs (see online supplementary table S1) in mice and humans. Regional expression of the GI appetite hormones CCK, PYY and GCG was also determined in mouse.

RNA was extracted from tissues using an RNeasy Mini kit (Qiagen). RNA quantity and quality was assessed using a NanoDrop. RT-PCR was performed as previously described.^16^ Primers (see online supplementary table S1) were designed and purchased from Geneworks (Adelaide, Australia); specificity was confirmed via product size by gel electrophoresis. Target gene expression was determined relative to endogenous controls using the comparative cycle threshold method normalised to glyceraldehyde 3-phosphatase dehydrogenase (GAPDH) or β-actin expression.^17^

### Ussing chamber experiments

Mouse and human colonic mucosa were divided into three 1×1 cm segments, and each segment was mounted in an Ussing flux chamber. The luminal surface was exposed to 10 mL nutrient solution, and the basolateral surface was exposed to 10 mL Krebs solution for 20 min. All solutions were carbogenated at 35–37°C. Colonic mucosa was then fixed in 4% paraformaldehyde overnight.

### Release assays

Collected biopsies (N=3–6) were incubated with 250 μL of carbogenated solutions of buffer (control) or nutrient in a 96-well plate for 15 min (5-HT assay) or 2 h (GLP-1 and PYY assay) at 37°C. A customised tissue culture medium with 4.4 mM L-glutamine and 6 mM glucose was used in order to ensure cells were healthy but not stimulated with nutrients. DPPIV inhibitor, PK 44 phosphatase (50 nM), was added to buffer in GLP-1 experiments, while monoamine oxidase inhibitor moclobemide (10 nM) and selective serotonin reuptake inhibitor fluoxetine (10 µM) were used in the 5-HT to prevent breakdown and reuptake. Following incubation, supernatants were collected and stored at −20°C. GLP-1 and PYY protein levels were quantitated using a human multiplex kit according to manufacturer's instructions (Milliplex MAP Multiplex assay, Merck Millipore), with ghrelin and gastric inhibitory polypeptide (GIP) included as comparators. 5-HT was measured using an ELISA (BA E-5900, Labor Diagnostika Nord). Addition of nutrients to control supernatants after the study did not affect readings, so their presence did not interfere with assays.

### Nutrient solutions

The luminal side of mouse colonic mucosa was exposed to 1 and 10 mmol/L phenylalanine (Phe) and tryptophan (Trp) while human ascending colon was exposed to 25 and 50 mmol/L Phe/Trp (selective activators of the CaSR). Lauric acid (LA, 12.5 and 25 mmol/L) was used as an agonist of GPR84 in separate experiments. We further investigated the intracellular pathway involved in activation and its relationship with mediator release by interference with G-protein coupling using pertussis toxin (PT, 200 ng/mL). Nutrients made up in Krebs solutions were either normal or high calcium (for Phe/Trp experiments). All solutions contained 124.05 mM NaCl (Sigma), 4.78 mM KCl (AnalR), 1.33 mM NaH_2_PO_4_ (Sigma), 2.44 mM MgSO_4_ (Sigma), 5.50 mM D-glucose (Sigma) and 25.00 mM NaHCO_3_ (Sigma) and carbogenated with 95% O_2_ and 5% CO_2_. Normal and high calcium solutions also contained 2.50 and 5.05 mM CaCl_2_, respectively.

### Immunohistochemistry

Immunolabelling for GPRC6A, T1R2, GPR93 and FFAR3 was assessed in mouse proximal jejunum and colon, with the latter three targets colocalised with CCK in jejunum and PYY/GLP-1 in colon (see online supplementary table S2). Colocalisation of CaSR and GLP-1/PYY/5-HT was also determined in human colon while pERK and pCamKII was performed in human and mouse Ussing chamber experiments (see online supplementary table S2). Briefly, 10 μm sections were washed with a blocking buffer, the primary antibody was applied (19 h, 4°C), tissues were then washed and incubated (60 min, room temperature) with species-specific Alexa Fluor conjugated secondary antibodies (1:200, Invitrogen). Sections were imaged as previously described.^16^ Immunopositive cells for each target were manually counted in each section and averaged over five fields of view.

### Statistical analysis

Data are expressed as mean±SEM. Statistical analysis was performed using one-way analysis of variance and Tukey's post hoc test (GraphPad Prism, V.5.02, GraphPad Software, Inc), with p<0.05 considered statistically significant. For the release assays, unpaired, one-tailed t tests were used and statistical significance was tested using Mann–Whitney post hoc test with p<0.05 defined as significant.

## Results

### Nutrient receptor distribution

**Figure 1:**
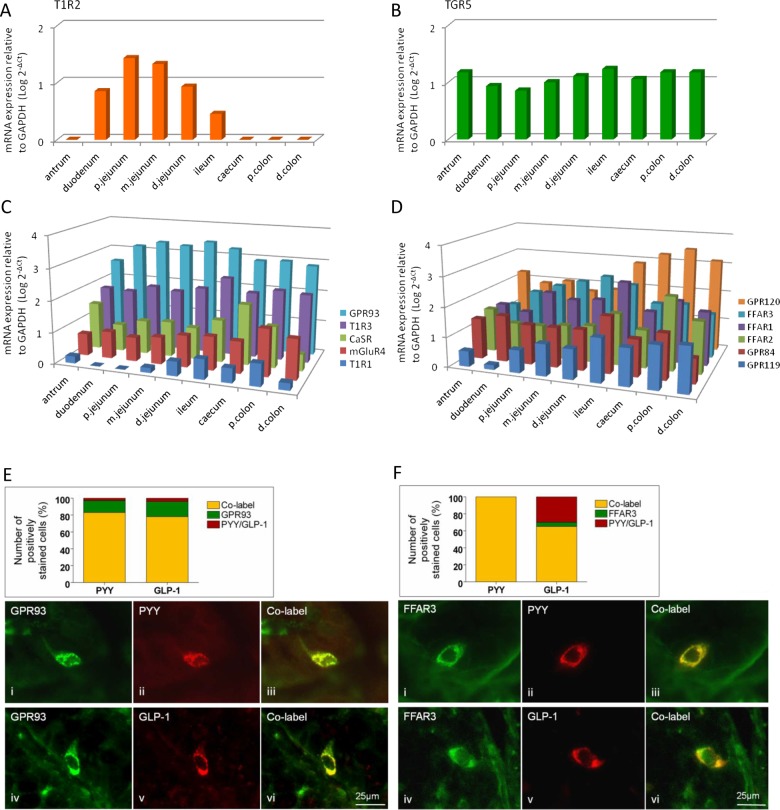
Regional expression in mouse GI tract of the G-protein coupled receptors (GPCR) for carbohydrate-sweet T1R2 (A), bile acids, TGR5 (B), amino acids, GPR93, T1R3, CaSR, mGluR4 and T1R1 (C), fatty acids GRP120, FFAR3, FFAR1, FFAR2, GPR84 and GPR119 (D), relative to GAPDH (n=7 for all expression studies). (E and F) Immunolabelling in mouse proximal colon for GPR93, FFAR3, GLP-1 and PYY in mouse proximal colon. Charts represent the proportion of individual GPCRs colocalised with cells expressing GLP-1 or PYY (yellow), those with GPCR alone (green) and those with hormone alone (red).

In order to answer the question of which receptors are present to sense nutrients in the GI tract, we first investigated the relative distribution of the major candidates throughout the mouse and human gut using quantitative measurement of their transcripts. Nutrient GPCRs were expressed throughout the mouse GI tract from stomach to colon, with the exception of the sweet taste receptor T1R2 that was detected only in the small intestine (figure 1). The bile acid sensor TGR5 did not reveal any regional specificity. The protein hydrolysate receptor GPR93 showed highest expression levels in the mid-intestine, peaking in the distal jejunum. The AA receptors CaSR, mGluR4 and T1R1 were more highly expressed distally, with peak expression evident in the caecum (CaSR) and proximal colon (mGluR4, T1R1). Expression of the T1R1 coreceptor T1R3 was highest in the ileum. The short chain fatty acid (SCFA) receptor FFAR2 was highly expressed in the proximal colon, while FFAR3 peaked in the distal jejunum. Both the medium CFA (MCFA) receptor GPR84 and the dual MCFA and long CFA (LCFA) receptor FFAR1 were highest in the ileum, while GPR120 levels peaked in the proximal colon. The oleoylethanolamide receptor GPR119 showed highest expression in the distal GI tract. As expected, cholecystokinin (CCK) transcript levels were highest in the small intestine, whereas glucagon (GCG) and PYY expression peaked in the proximal and distal colon, respectively (see online supplementary figure S1).

**Figure 2:**
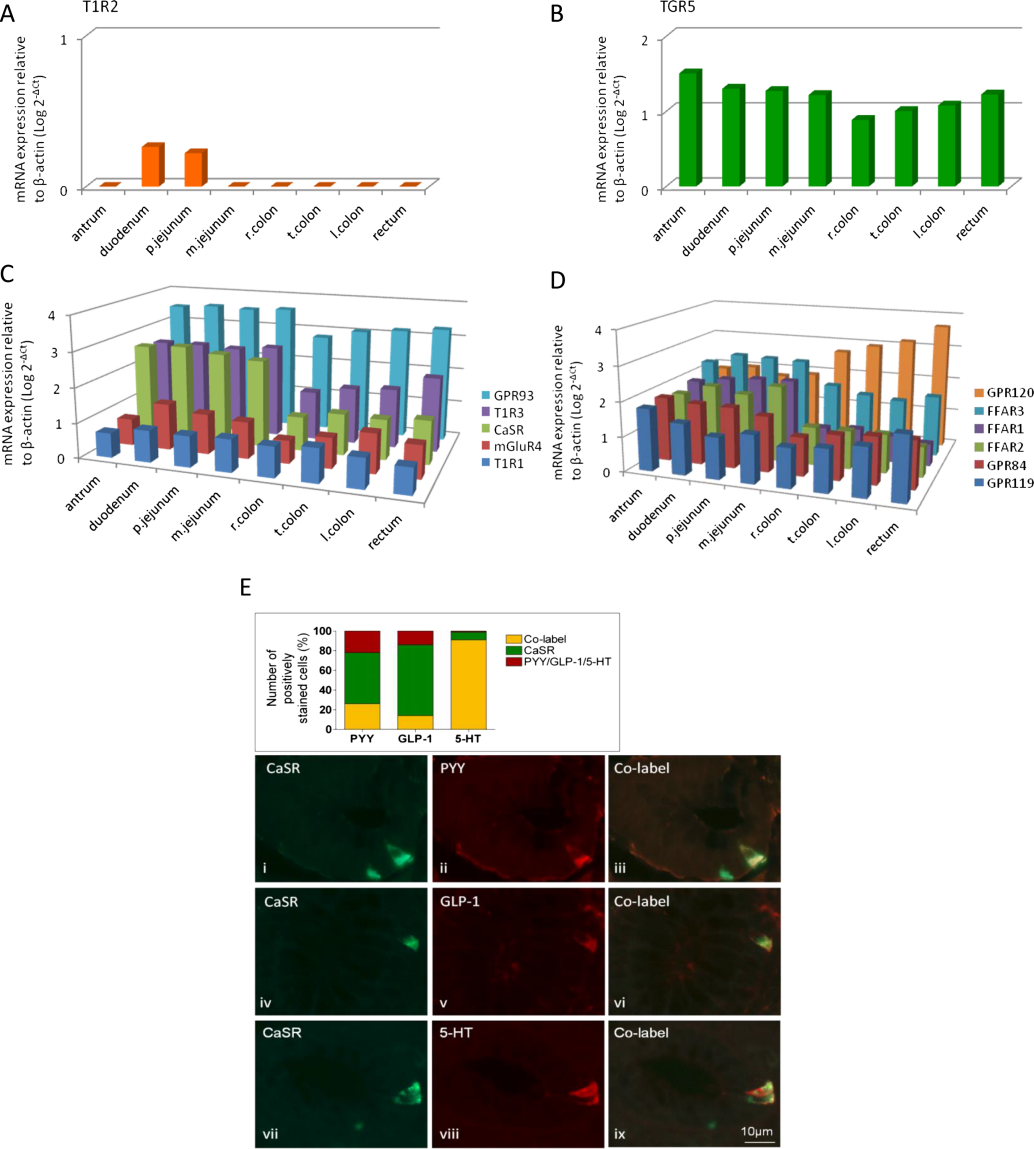
Regional expression in human GI tract of the G-protein coupled receptors (GPCR) for carbohydrate-sweet T1R2 (A), bile acids, TGR5 (B), amino acids, GPR93, T1R3, CaSR, mGluR4, T1R1 (C), fatty acids GRP120, FFAR3, FFAR1, FFAR2, GPR84 and GPR119 (D) relative to β-actin (n=6 for all expression studies). (E) Immunolabelling in human proximal colon for CaSR, GLP-1, PYY and 5-HT. Chart represents the proportion of individual cells colocalised with PYY, GLP-1 or 5-HT with and without CaSR.

Since the diet and food intake behaviour of humans and mice differ considerably, it was important to determine whether patterns of expression were conserved between these species. This was generally the case since all nutrient GPCRs were expressed in biopsies collected over the length of the human GI tract, with the exception of T1R2, which was only detected within the small intestine of both species (figure 2). GPR93 was the most highly expressed receptor in the small intestinal mucosa in mice and humans, while the LCFA receptor GRP120 was highest in the large intestine. GPR93 and the SCFA receptor FFAR2 were also highly expressed in the large intestine in both species.

### GPCRs within EECs

Immunolabelling for T1R2, GPR93 and FFAR3 was assessed in mice in combination with CCK labelling in the mouse jejunum, and with GLP-1 and PYY in the colon, since mRNA for these mediators was predominant in these locations (see online supplementary figure S1). T1R2 did not colocalise with CCK, whereas 32% of FFAR3-positive cells were CCK-immunoreactive (IR) (see online supplementary figure S2). Colonic L-cells were IR for GLP-1 and PYY, and were frequently associated with GPR93 and FFAR3 labelling (figure 1E, F). Similar colocalisation of GLP-1 and PYY was found in the ascending colon in humans with 69% overlap of cells labelled with each individually. As our interest was particularly in the role of the distal gut in nutrient sensing, colocalisation of CaSR with the major EECs mediators PYY, GLP-1 and 5-HT was examined using double-staining immunolabelling. The data in figure 2E indicate that although a significant population of L-cells (GLP-1 and/or PYY-IR) expressed CaSR, the majority of CaSR expressing cells did not colocalise. Instead, they were frequently associated with 5-HT-IR (91%), suggesting that CaSR signalling may act to preferentially release 5-HT in the human ascending colon. The greatest number of CaSR-IR cells in humans was found within the antrum (see online supplementary figure S3), consistent with its established role in AA-evoked gastrin release,^18^ followed by the ileum and colon. The predominance of 5-HT colocalisation with CaSR in colon was not seen in mouse (see online supplementary figure S4), suggesting species differences.

### Downstream pathways activated by CaSR

**Figure 3:**
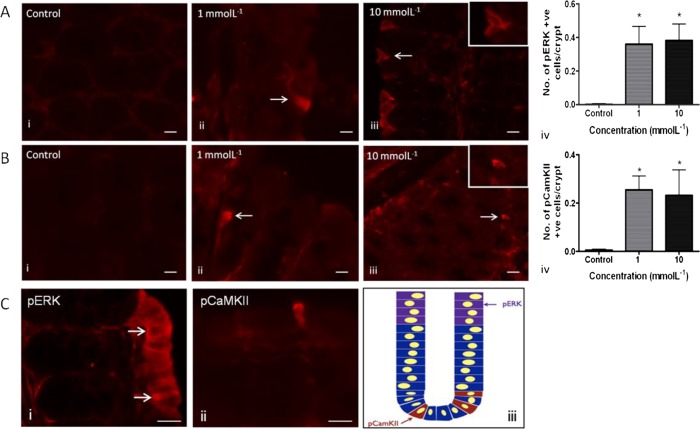
Effects of phenylalanine and tryptophan (1 and 10 mmol/L) on enzyme phosphorylation in sheets of mouse proximal colonic mucosa. There was a significant increase in the number of pERK-IR (A) and pCamKII-IR (B) cells following stimulation at both concentrations (*p<0.05) compared with control. pERK-IR cells were commonly observed in groups towards the apical aspect of mucosal villi. Within pERK-positive cell groups, one or two cells often stained more positively than surrounding IR cells (C (i), arrows). Comparatively fewer pERK-positive cells appeared in crypts. The opposite was true for pCamKII-IR cells (C (ii) and schematic in (iii)). Insets show 40× magnification of activated cells. Scale bar 15 μm.

As we found expression of AA sensing receptor mRNA in mouse and human distal GI tract, and expression of CaSR protein in the human colon, we explored the functional role of this receptor in activation of appetite regulation pathways. The cell activation markers phospho-extracellular signal-regulated kinase (pERK) and pCaMKII were used as markers of general cellular activation. In mouse proximal colon, in response to stimulation with the CaSR agonists phenylalanine and tryptophan (Phe/Trp), we observed increased pERK (figure 3A) and pCaMKII (figure 3B) expression compared with buffer control. These markers revealed different patterns of activation of cell groups (pERK; figure 3C (i)) or individual cells (pCAMKII; figure 3C (ii)). This pattern of pERK activation may suggest entrainment of cells neighbouring some types of EECs, but not others. In order to confirm that these effects were receptor-mediated, we repeated the experiments with the CaSR selective agonist cinacalcet (1 µM),^19^ which showed similar induction of activation markers to Phe/Trp (see online supplementary figure S5).

**Figure 4:**
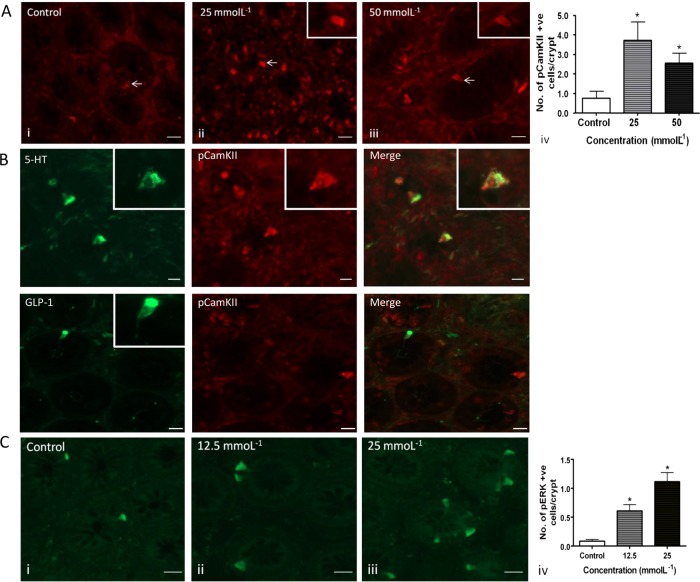
(A) Human ascending colonic mucosa was stimulated with 25 and 50 mmol/L Phe/Trp or high calcium Krebs as control. There was a significant increase in the number of pCamKII-IR cells following stimulation at both concentrations (*p<0.05) compared with control. pERK-IR was not induced (not shown). Scale bar 15 μm. (B) In human colon, Phe/trp at 25 mmol/L resulted in specific activation of 5-HT-IR pCaMKII cells, while GLP-1 cells were not found to colocalise with pCamKII. 5-HT-IR and GLP-IR were found predominantly in the basolateral membrane (insets), suggesting that the peptide was located in vesicles in anticipation of release following CaSR stimulation. Scale bar 15 μm. (C) Effect of lauric acid (12.5 and 25 mmol/L) on pERK-IR in sheets of human proximal colonic mucosa. There was a concentration-dependent increase in the number of IR cells with increasing concentrations (*p<0.05) compared with control. Scale bar 15 μm.

The effects of CaSR stimulation were also studied in the human proximal colon. pCaMKII induction was concentration-dependent in response to Phe/Trp (figure 4A), whereas in contrast to the mouse, we never observed pERK induction above baseline. Since CaSR was present mainly in 5-HT-containing cells rather than L-cells (figure 2E), we investigated the presence of 5-HT or GLP-1 in the activated cells. This experiment confirmed the association of the CaSR with 5-HT-containing cells (figure 4B). Following luminal exposure to 25 mmol/L Phe/Trp, four times more 5-HT-IR cells exhibited colocalised pCamKII-IR (42±11%) than under control conditions (10±5%, p<0.01, n=10). GLP-1 cells also showed CamKII activation, although to a lesser extent (29±5% p<0.01).

As pERK was induced in mouse but not human tissue, this raised the possibility that the pERK pathway was species specific. In order to determine whether pERK was in fact inducible by nutrients in human tissue, we investigated the effect of LA, which is known to be a potent releaser of hormones other than 5-HT.^20^ There was indeed a concentration-dependent increase in the number of pERK-IR cells in the human proximal colon after exposure to LA (figure 4C), which we showed to occur in both 5-HT-containing and L-cells (see online supplementary figure S6).

**Figure 5:**
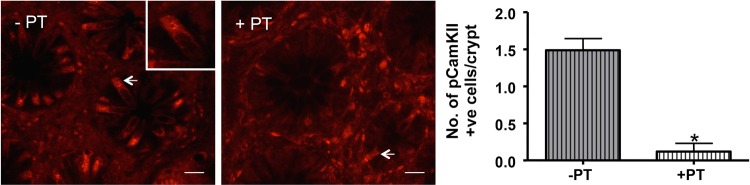
Phe/trp stimulation of human ascending colon mucosa pretreated with pertussis toxin (+PT) resulted in a significant decrease in the number of pCamKII-IR cells/crypt (arrows) compared with untreated controls (−PT, *p<0.05). Scale bar 20 µm.

CaSR is coupled via Gαq and Gαi/o G-proteins, so we wanted to confirm such a mechanism was involved in the response of human EEC to Phe/Trp. Therefore, PT was used to block activation of Gi/o and thus downstream activation. Pretreatment with PT resulted in a significant decrease in the number of pCAMKII-activated cells/crypt (figure 5), confirming G-protein involvement. Therefore, with regards to CaSR coupling in human, it appears to activate via G-proteins, which are in turn coupled with either pCAMKII in 5-HT-containing ECs, or a different mechanism in L-cells, but not to pERK.

### Mediator release

**Figure 6:**
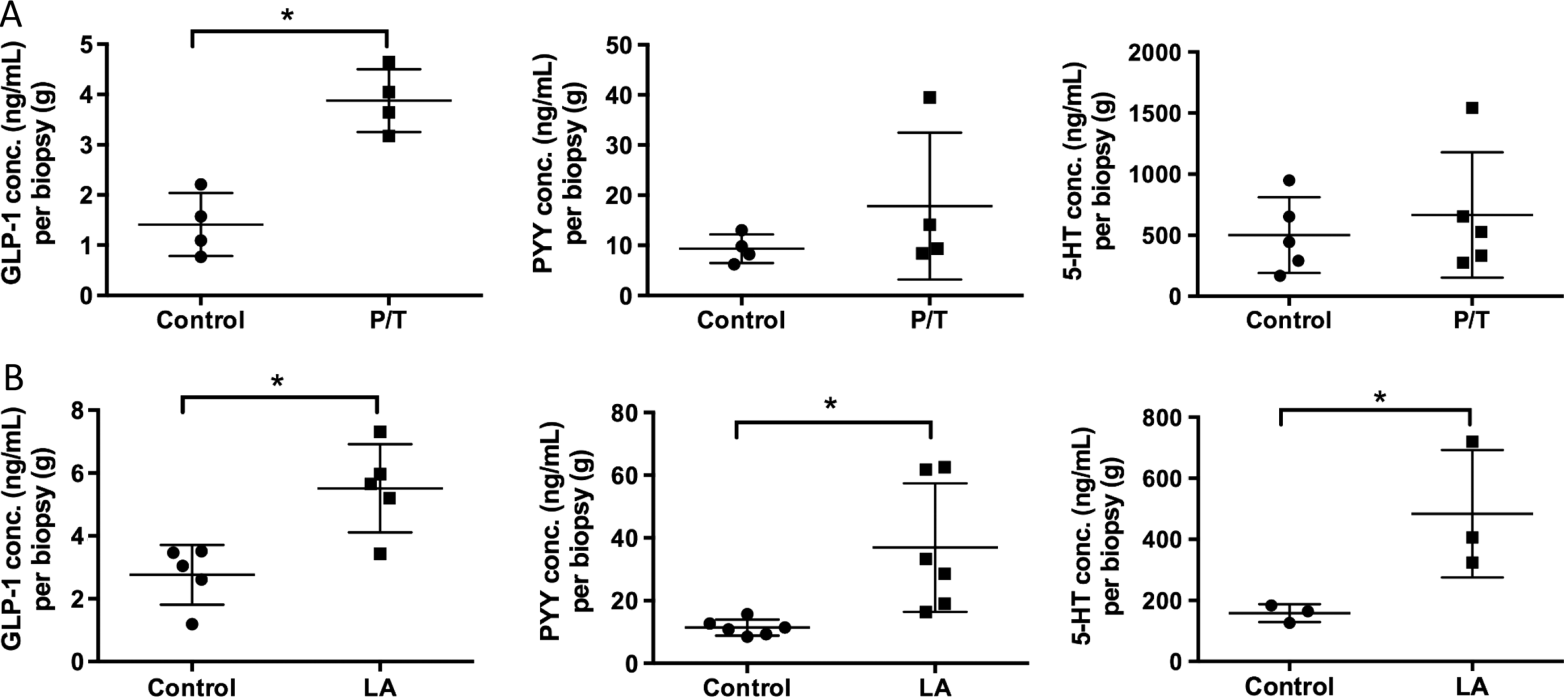
Stimulation with nutrients phenylalanine/tryptophan (P/T) and lauric acid (LA) induces release of appetite regulating hormones in the human proximal colon. (A) Colonic biopsies stimulated with 50 mM P/T induce a significant increase in GLP-1 release but not PYY or 5-HT. (B) 25 mM LA significantly increased release of GLP-1, PYY and 5-HT compared with buffer control (*p<0.05).

As we found activation of EEC in mouse and human distal GI tract, and expression of CaSR and GPR84 in the human colon, we explored the functional consequence of receptor activation by measuring release of the three mediators that we examined immunohistochemically. LA (which activates GPR84 most potently) evoked significantly more release of all three mediators compared with control, whereas Phe/Trp (which activate CaSR most potently) evoked increased release only of GLP-1, with trends towards increases of PYY and 5-HT (figure 6). Neither of these stimuli evoked release of GIP or ghrelin (data not shown).

## Discussion

In this study, we have gained insight into which receptors mediate satiating actions of nutrients in the GI tract. Throughout the mouse and the human GI tract, AA and FA receptors were expressed from stomach to colon, with a high level of species similarity and conservation of expression in the distal gut. We found specific colocalisation of receptors with specific EEC mediators, for example, calcium-sensing receptor and 5-HT, suggesting a fine-tuning of EEC for particular stimuli. This was evident in functional studies: in human colon, AAs preferentially activate one intracellular signalling pathway (pCamKII) in 5-HT containing cells, whereas fatty acid activates another (p-ERK) in GLP-1- and/or PYY-containing cells.

As previously shown, the EEC mediator cholecystokinin (CCK) was found mainly in the small intestine, whereas glucagon (GCG) and PYY gene expression peaked in the colon. Carbohydrate in the intestinal lumen is not associated with CCK release, whereas protein and fat generally is. Correspondingly we found T1R2 did not colocalise with CCK, whereas 32% of FFAR3-positive cells were CCK-IR. Colonic L-cells were IR for GLP-1 and PYY, and were frequently associated with GPR93 and FFAR3 labelling, again suggesting a link with protein and fat sensing. Although a significant population of L-cells (GLP-1 and/or PYY-IR) expressed CaSR, the majority of CaSR-expressing cells did not colocalise. Instead, they were almost exclusively associated with 5-HT-IR. These observations are consistent with overlapping lineages of EECs, with ECs having a largely non-overlapping phenotype to others.^21^

To determine the functional role of nutrient receptors in activation of appetite regulation pathways, we looked for markers of cell activation. Extracellular signal-regulated kinase (ERK) becomes phosphorylated as a result of a cascade of activation by GPCRs coupled via Gq and Ras-Raf proteins in many systems and interacts with pathways of secretion, gene expression and cell metabolism.^22^ Calmodulin kinase (CaMKII) is activated by increases in intracellular calcium normally associated with release from intracellular stores following Gq activation of phospholipase C and stimulation of ryanodine receptors on endoplasmic reticulum.^23^ This pathway is activated alongside the intracellular calcium-dependent docking of secretory vesicles. Therefore, these two intracellular pathways may have convergent and divergent effects, and share the feature of Gq-protein activation.^23^ The majority of nutrient receptors are coupled via this G-protein, so the markers we chose to investigate are likely to reveal alternative downstream cascades. pCaMKII has been previously shown to be a suitable marker for activated EEC in stimulated intact mucosa. pERK is well established as a component of synaptic neural activation.^24^

In mouse proximal colon, in response to stimulation with the CaSR agonists phenylalanine and tryptophan, both markers were induced but revealed different patterns of activation. pERK was induced in cell groups in several cases, whereas pCAMKII was activated only in individual cells. The pattern of pERK activation suggests entrainment of cells neighbouring some types of EEC, but not others. We speculate that this may be a mechanism of paracrine control of absorptive, immune or proliferative function of colonocytes. The effects of CaSR stimulation were different in the human proximal colon. pCaMKII was induced concentration-dependently in response to Phe/Trp, whereas in contrast to the mouse, we did not observe pERK induction. It was therefore important to determine whether a GPCR mechanism was still involved. This was confirmed using PT to block activation of G-proteins. These data raised the possibility that pERK was a marker of nutrient receptor activation only in the mouse, so we investigated responses to another nutrient for which receptors are present in the human colon. Therefore, we incubated human tissue with LA (12.5 and 25 mM), which is an agonist of GPR84 and evokes PYY and GLP-1 release.^20^ This evoked powerful pERK responses in a large number of EECs, indicating that there are different pathways involved in aromatic AA and MCFA sensing in different cell types. Correspondingly, LA evoked potent release of all three EEC mediators.

Since CaSR was present mainly in 5-HT-containing cells rather than L-cells (figure 2E), we investigated the presence of 5-HT or GLP-1 in the activated cells. This experiment confirmed the association of the Phe/Trp-sensing receptor CaSR mainly with 5-HT-containing cells (figure 3D). Therefore, with regards to CaSR coupling, it appears to activate human EE cells via G-proteins, which are in turn coupled with pCAMKII in 5-HT-containing ECs, and may involve in addition a different mechanism in L-cells, but one that is independent from pERK.

### Conclusions

We conclude that the distal gut of humans and mice is extensively equipped with sensors for products of fat and protein digestion, and that these associate with specific signalling pathways, two of which have been shown here. These are in turn associated with the release of specific mediators. We have uncovered a role for 5-HT in the response to luminal aromatic AAs, which may contribute to metabolic and behavioural responses to exposure of the distal gut to a meal. It is already known that oral nutrient preloads reduce subsequent food intake^15^ and that bypass surgery reduces food intake by shunting nutrient to the distal gut. By refining nutrient preloads and formulating them to target the distal gut, we expect to develop a successful weight loss and antidiabetic strategy prior to and possibly in place of bypass surgery.

## Supplementary Material

Web supplement
