# Supplementary material for: Mechanisms of activation of mouse and human enteroendocrine cells by nutrients
Source: Gut. 2014 Jul 11;64(4):618–26. doi: 10.1136/gutjnl-2014-306834 (PMC4392230; doi:10.1136/gutjnl-2014-306834)
Supplement: Web supplement [file gutjnl-2014-306834-s1.pdf]

Supplementary Data. Symonds et al. Gut

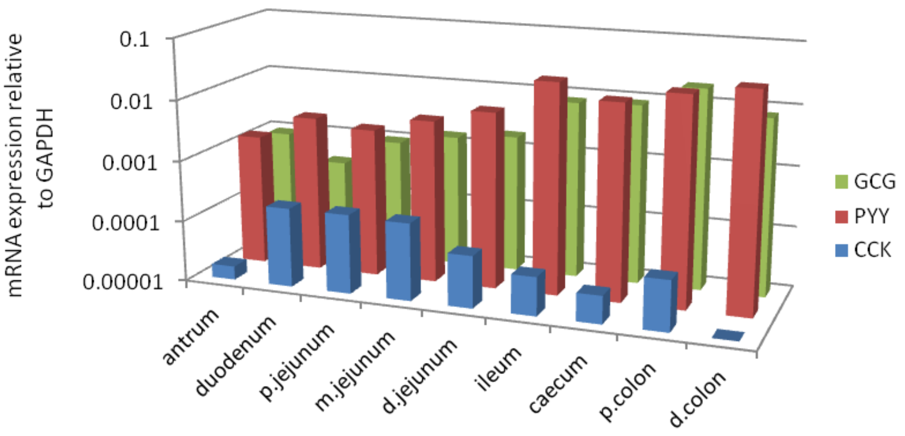

Fig S1. Regional expression of cholecystokinin (CCK), peptide YY (PYY) and proglucagon (GCG) in mouse mucosa. Data are expressed relative to GAPDH expression (n = 7).

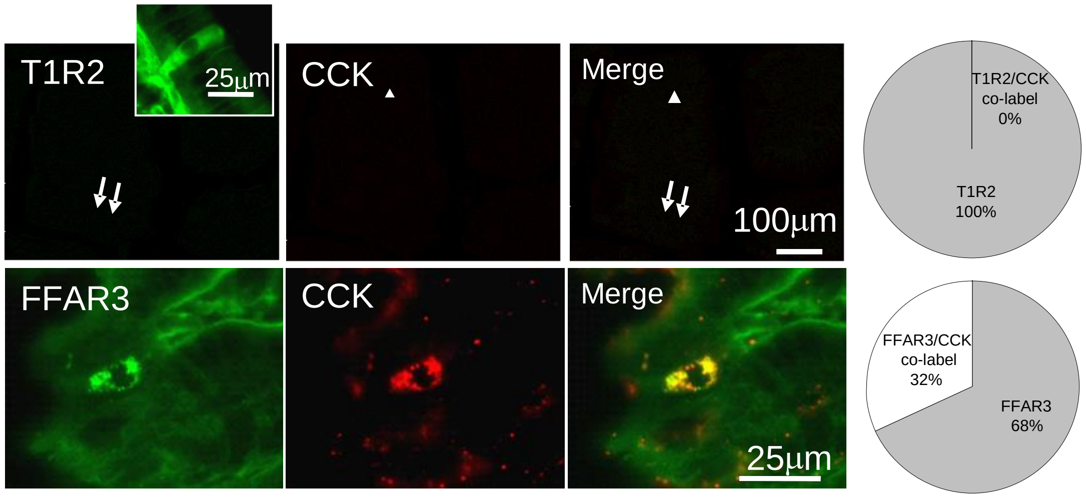

Fig S 2. Double immunolabelling in mouse proximal jejunum for T1R2 and CCK T1R2, and FFAR3 and CCK, and pie charts showing extent of co-localization. Inset shows higher magnification of T1R2 labelling.

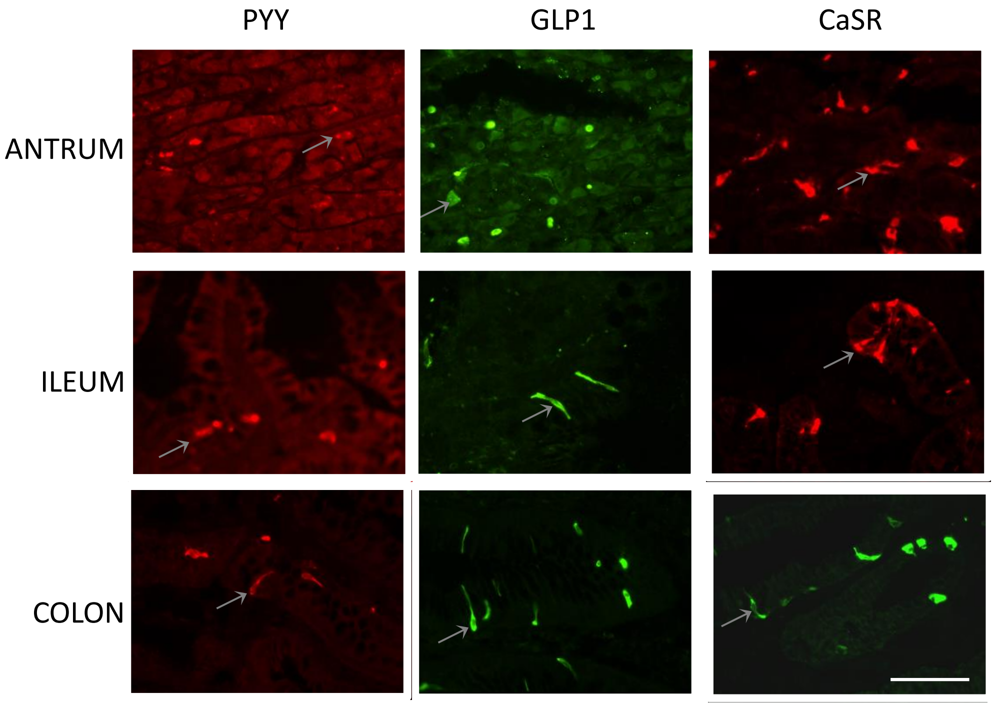

Fig S 3. CaSR-immunoreactive (IR) cells in human antrum, ileum and colon. GLP-1 and PYY-IR is shown for comparison. CaSR positive cells were generally found in clusters and cells that were adjacent were often observed. These cells were found mostly within the deeper layers of gastric glands but were also seen close to the surface around gastric pits. They were more compact and less elongated than those in the intestine, which may suggest a closed phenotype. CaSR-IR cells in the ileum and colon were found superficially within the villi and crypts, respectively. CaSR-IR cells of the colon had similar morphology to those of the ileum although there were significantly fewer CaSR-IR cells in the colon compared to the antrum or ileum. Scale bar = 20 μm

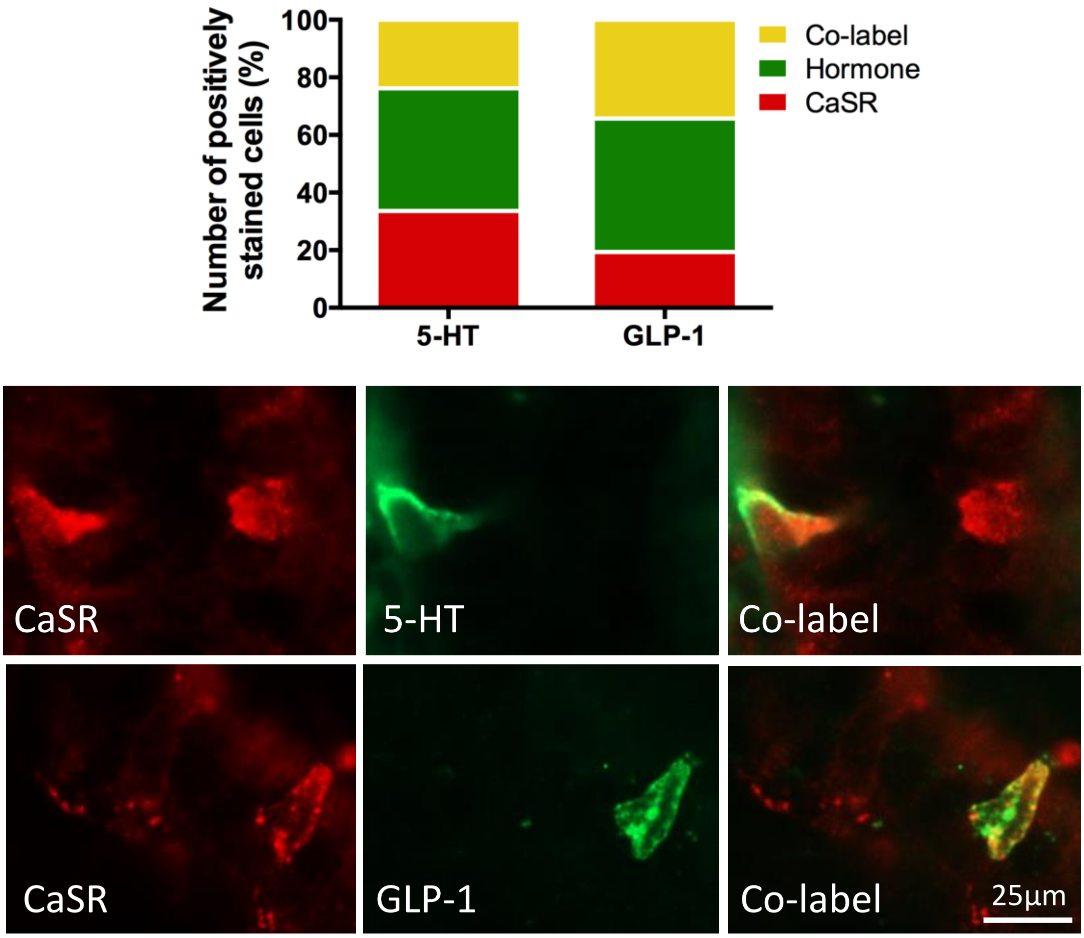

Fig S 4. CaSR-immunoreactive (IR) cells in mouse proximal colon, and colocalisation with 5-HT and GLP-1. Proportions are shown in the graph of co-labelled (yellow), 5-HT or GLP-1 alone (green) or CaSR alone (red) cells. Scale bar = 25 µm

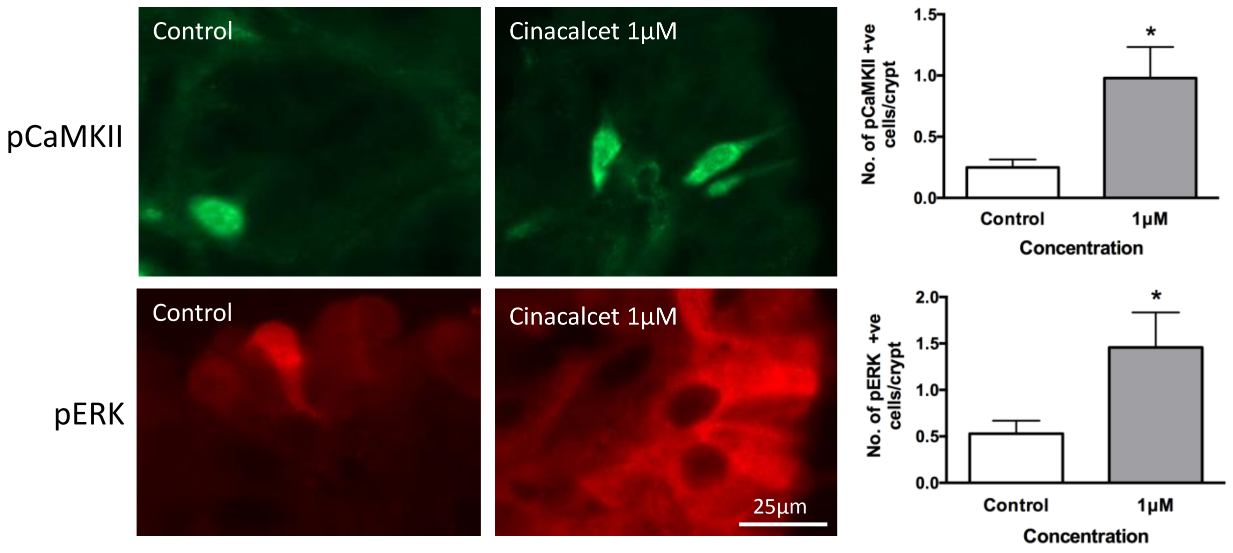

Fig S 5. Cells labelled for pCaMKII or pERK in mouse colon in either control conditions, or after exposure to Cinacalcet. Significant increases were seen in both markers (* *P*<0.05, t-test). Scale bar = 25 µm

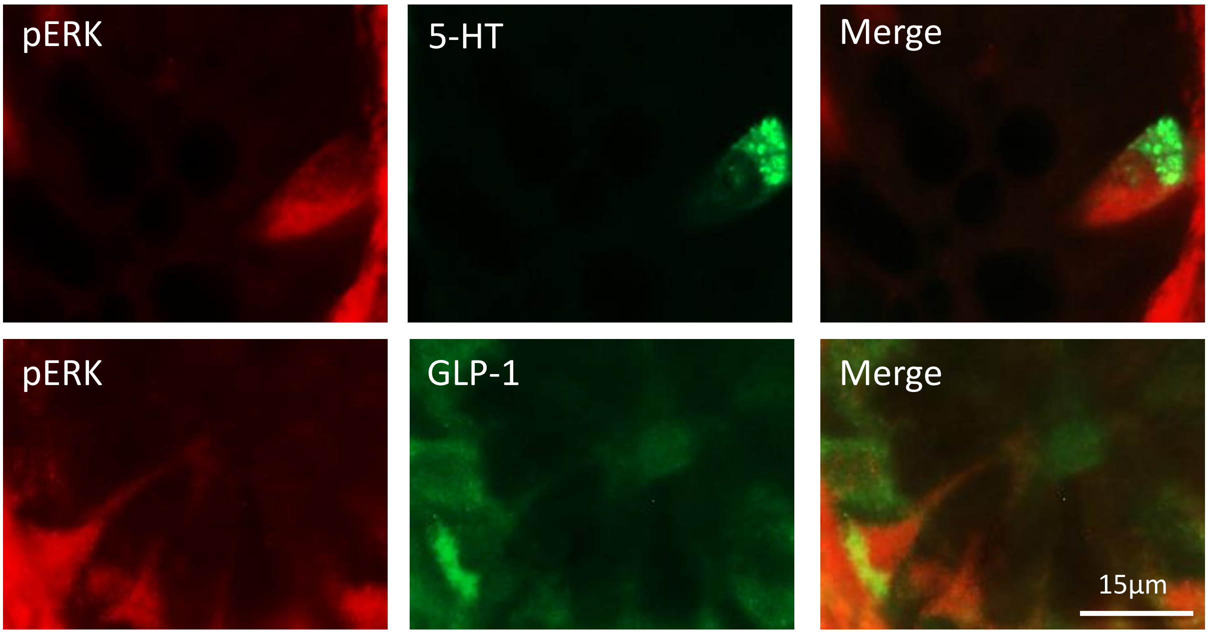

Fig S 6. Cells labelled for pERK in human ascending colon after exposure to Lauric acid (25mM). Activation was seen in both 5-HT and GLP-1 – immunoreactive cells. Scale bar = 15 µm

SUPPLMENTARY TABLE 2. Primer sequences used for RT-PCR.

**Mouse primers**		
	**Forward primer (5' to 3')**	**Reverse primer (5' to 3')**
FFAR3	CGCAAGAGGATAATGGGG	GAGTTGAGGGTGCTGAGGAG
FFAR2	GAACTTCACCCAAGAGCAGC	TAAGAAGCGTCACAACAGCC
GPR84	AGGTGACCCGTATGTGCTTC	TGTTGAGCCAGGTGAGGTTG
GPR119	ATTCCAGCAGACCACCTACC	GCACAGAGGCAATCTTGAGC
GPR120	TGCCGGGACTGGTCATT	GTTGGGACACTCGGATCTG
T1R1	GATGCCCCTGGTCAGCTATG	GCAGCCGCACTATGACTTCC
T1R3	CATTACAGGCAAGTTCTTCAGC	TCACTCCCTAAGGCGGC
CaSR	GCATCAGGTATAACTTCCGTGG	TTGGAGACGGTGTTACAGGTG
mGluR4	CGTCCAATGCCAGAGCC	TTGGGAAGAATGGTGACTGC
GPRC6A	GCCACAGGTGAGTTATGAATCC	CCAATCCAGTTCCATCCCG
GPR93	GCTGTCGTCTATTCGTCTGGC	CGAAGCACAGCAGGAAGATG
TGR5	CTGCTGGCTGCTTCTTCC	CACTGCCATGTAGCGTTCC
PYY	CAATGCTGCTAATCCTGCTCG	TTTGGAGAACAGAGCTGCGG
GCG	GGAACCGGAACAACATTGC	CCTTCCTCGGCCTTTCAC
CCK	AGAGCGGCGTATGTCTGTGC	GATGTATCGCGCTAGCAGTGC
T1R2	(QT00142639, Qiagen)	
FFAR1	(QT00308833, Qiagen)	
GAPDH	(QT01658692, Qiagen)	
**Human primers**		
FFAR1	CAACGTCCTGGCCATCCG	GTCGGAGCAGCCCAGGTTC
FFAR3	ATCACTGGTTCGTCTTCTCGG	AGGCGGTCAGGTTGAGCA
FFAR2	CAAGATCATCGAGGCTGC	AAGCCACTCCCAGGTAGC
GPR84	AATGTGCTCACCCTACTGGC	GCGTGCAGTAGAGGAGATCAG
GPR120	TCTCTGCGTCTTCTTCCG	GTGGGCCAAATCAGTGTG
T1R1	GAGATAAACAACTCCACGGC	CACTCTCAGCGTGGCATAC
T1R3	TATAGCGTGGCCCAGGC	GTTCTCCAGGAGCTGCCAG
CaSR	TTCCCCAGGTCAGTTATGC	AATTGTGCCCACCCAGTT
GPR93	GACGAGCTGTGGAAAGGC	CGAAGCACAGCAGGAAGAT
T1R2	(QT01026508, Qiagen)	
mGluR4	(QT00060396, Qiagen)	

GPR119	(QT01030848, Qiagen)
TGR5	(QT00209594, Qiagen)
βactin	(QT01680476, Qiagen)

SUPPLEMENTARY TABLE 2. Antibodies used for immunohistochemistry.

Protein	Antibody details	Dilution
T1R2	rabbit polyclonal (SC-50306, Santa Cruz Biotechnology)	1:200
GPR93	rabbit polyclonal (SC-135237, Santa Cruz Biotechnology)	1:100
GPRC6A	rabbit polyclonal (SC-98332, Santa Cruz Biotechnology)	1:50
CaSR	mouse monoclonal (AB-19347, Abcam)	1:200
FFAR3	rabbit polyclonal (SC-98332, Santa Cruz Biotechnology)	1:80
CCK	goat polyclonal (SC-21616, Santa Cruz Biotechnology)	1:200
GLP-1	goat polyclonal (SC-7782, Santa Cruz Biotechnology)	1:200
PYY (mouse)	guinea pig polyclonal (PAB17185, Abnova)	1:6000
PYY (human)	rabbit polyclonal (P3285-10, US Biological)	1:200
5-HT	mouse polyclonal (5HT-H209, Dako)	1:200
pERK	rabbit monoclonal (4370, Cell Signalling)	1:200
pCamKII	rabbit polyclonal (V1111, Promega)	1:100
